# The flavonoid agathisflavone modulates the microglial neuroinflammatory response and enhances remyelination

**DOI:** 10.1016/j.phrs.2020.104997

**Published:** 2020-09

**Authors:** Monique Marylin Alves de Almeida, Francesca Pieropan, Larissa de Mattos Oliveira, Manoelito Coelho dos Santos Junior, Jorge Mauricio David, Juceni Pereira David, Victor Diógenes A. da Silva, Cleide dos Santos Souza, Silvia Lima Costa, Arthur Morgan Butt

**Affiliations:** a Department of Biochemistry and Biophysics, Institute of Health Sciences, Federal University of Bahia, Brazil; b School of Pharmacy and Biomedical Sciences, University of Portsmouth, United Kingdom; c Department of Health, State University of Feira de Santana, Brazil; d Department of General and Inorganic Chemistry, Institute of Chemistry, Federal University of Bahia, Brazil; e Department of Medication, Faculty of Pharmacy, Federal University of Bahia, Brazil; f Sheffield Institute for Translational Neuroscience, University of Sheffield, United Kingdom

**Keywords:** Remyelination, Neuroinflammation, Microglia, Flavonoids, Agathisflavone, Estrogen receptors

## Abstract

Myelin loss is the hallmark of the demyelinating disease multiple sclerosis (MS) and plays a significant role in multiple neurodegenerative diseases. A common factor in all neuropathologies is the central role of microglia, the intrinsic immune cells of the central nervous system (CNS). Microglia are activated in pathology and can have both pro- and anti-inflammatory functions. Here, we examined the effects of the flavonoid agathisflavone on microglia and remyelination in the cerebellar slice model following lysolecithin induced demyelination. Notably, agathisflavone enhances remyelination and alters microglial activation state, as determined by their morphology and cytokine profile. Furthermore, these effects of agathisflavone on remyelination and microglial activation were inhibited by blockade of estrogen receptor α. Thus, our results identify agathisflavone as a novel compound that may act via ER to regulate microglial activation and enhance remyelination and repair.

## Highlights

• Agathisflavone enhances remyelination through modulating neuroinflammation.
• Agathisflavone induces a polarization in microglia from an M1 to an M2-like phenotype.
• Agathisflavone interacts with the nuclear receptors RAR, RXRα, RXRγ, ERα, and ERβ.
• Remyelination effect of agathisflavone was via ER, with a greater effect of ERα.

## Graphical abstract

**Figure:**
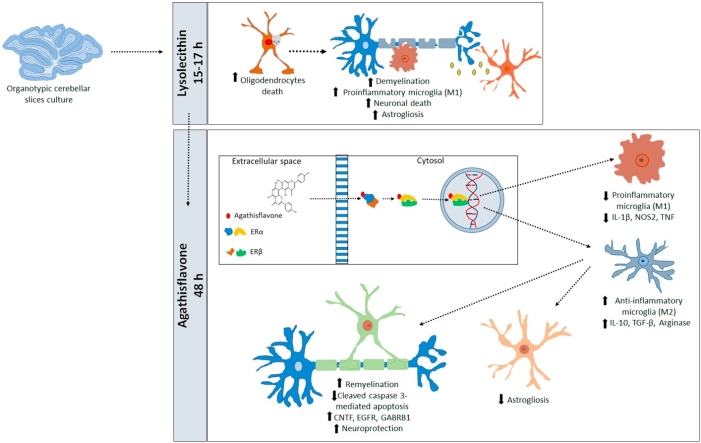

## 1 Introduction

Oligodendrocytes are central nervous system (CNS) glial cells responsible for producing myelin, the fatty insulation around axons that is essential for maintaining axonal integrity and to ensure the rapid transmission of action potentials [1]. The loss of myelin has devastating effects on CNS function and ultimately leads to neuronal degeneration, which are hallmarks of the demyelinating disease multiple sclerosis (MS) and other neuropathologies [2]. Notably, the CNS contains a significant population of oligodendrocyte precursor cells (OPCs), which are responsible for oligodendrocyte regeneration and are therapeutic targets in new strategies for stimulating remyelination and repair [3].

Microglia are the intrinsic immune cells of the CNS and respond to neuropathology by a process termed activation [4]. Microglia exhibit multiple states of activation and a high degree of heterogeneity. Based on expression of specific proteins and cytokines/chemokines, two distinct polarized microglial phenotypes have been described in the literature: pro-inflammatory M1 microglia and anti-inflammatory M2 microglia. However, there is now an abundance of evidence from microglial transcriptomic and proteomic profiles that characterizing microglia as being exclusively in an M1 or M2 state is over simplistic [5]. Nonetheless, the M1/M2 terminology remains in use as an indicator of microglial function and both polarized states are considered crucial to different stages in the pathogenesis of demyelination and remyelination [6]. Indeed, it has been reported that ‘M1’ microglia predominate during demyelination, and a switch to an ‘M2’ profile is necessary for efficient remyelination and repair [7]. Microglia with an ‘M2-like’ phenotype actively and more efficiently clear myelin debris than ‘M1-like’ microglia and secrete several trophic factors that promote neurogenesis and oligodendrocyte differentiation [8,9]. Therefore, although the classification of M1/M2 microglia is an over simplification, it is evident that modulating the inflammatory functions of microglia is an important strategy towards boosting efficient repair and remyelination [10].

Flavonoids are a heterogeneous group of polyphenolic bioactive compounds derived from plants that have prominent anti-inflammatory activity [11]. Agathisflavone is a flavonoid derived from the Brazilian plant *Poincianella pyramidalis* (Tul.), which we have previously shown to have neuroprotective and neuromodulatory effects *in vitro* [12,13]. Previously, we postulated that agathisflavone may act via estrogen receptors (ER) and retinoic acid receptors (RAR) [14], which are pharmacological targets for the treatment of demyelinating conditions [15,16]. Estrogen signalling is recognised to promote remyelination through its regulation of neuroinflammation [17], whilst retinoic acid signalling has been shown to promote OPC differentiation into myelinating oligodendrocytes [18].

Here, we show that in the *ex vivo* lysolecithin (LPC) model of demyelination in cerebellar slices, agathisflavone induces polarization of microglia from an M1- to an M2-like phenotype, while enhancing oligodendrocyte differentiation and promoting remyelination. Moreover, we demonstrate for the first time that estrogen receptor activation is required for agathisflavone induced remyelination.

## 2 Materials and methods

### 2.1 Animals and tissue

Mice (males and females) were killed humanely by cervical dislocation, in accordance with the UK Animals (Scientific Procedures) Act, 1986 and with the University of Portsmouth Ethics Committee. Mice aged postnatally (P)10–12 from different backgrounds were used throughout this study. Mice belonging to the C57BL/6 background were used for protein and gene expression quantification RT -RT-qPCR and immunohistochemistry). Transgenic mice in which the expression of the Enhanced Green Fluorescent Protein (EGFP) is under the control of the SOX10 or the Glial Fibrillary Acidic Protein (GFAP) genes were used to identify oligodendrocytes and their precursors (OL/OPC) and astrocytes respectively (gifts from William Richardson, UCL, UK and Frank Kirchhoff, University of Saarland, Germany, respectively).

### 2.2 Organotypic cerebellar cultures

To analyze the effects of agathisflavone, we used *ex vivo* organotypic cerebellar slices and the L-α-Lysophosphatidylcholine (LPC) model of demyelination that have been previously described and published [[19], [20], [21], [22]]. In brief, cerebella from P10-12 mice were dissected into oxygenated ice-cold dissecting solution containing (in mM): 25.95 NaHCO_3_, 1.39 NaH_2_PO_4_, 10 glucose, 124 NaCl, 2.95 KCl, 10 MgCl_2_, 2 CaCl_2_, 1 MgSO_4,_ 1000 units/mL penicillin/streptomycin), and 300 μm cerebellar parasagittal slices were cut using a vibrating microtome 5100mz (Campden Instruments LTD). Slices were then transferred to a membrane insert (Millipore, 30 mm diameter, pore size 0.4 μm) and cultured using an interface method, with 1 ml of serum-based medium composed of 50% Minimum Essential Medium with Glutamax-1 (MEM), 23% Earle’s Balanced Salt Solution (EBSS), D-glucose (0.13 mg/mLl), 1% penicillin-streptomycin, and 25% horse serum (Gibco Invitrogen). Slices were maintained at 37 °C, under standard conditions (95% O_2_/5% CO_2_) for 7 days *in vitro* (DIV), at which timepoint oligodendrocytes differentiate and there is significant myelination [19].

### 2.3 Agents and treatments

The flavonoid agathisflavone (FAB) was extracted from *Poincianella pyramidalis* (Tul.) as previously described [23] and stored protected from light at −20 °C at a stock concentration of 10 mM in dimethyl sulfoxide (DMSO; Sigma Chemical Co). After 7 DIV, slices were treated for 15–17 h with medium containing LPC (0.5 mg/mL, Sigma, L4129), after which LPC-medium was removed and replaced with medium containing either agathisflavone at the concentrations of 5 or 10 μM, or 0.1% DMSO vehicle (LPC + DMSO condition), for a further 2DIV; concentrations of agathisflavone used were based on previous studies by our group. The effects of LPC were compared to slices that were maintained in normal medium during 7DIV (controls), which displayed normal myelination and cellular integrity. To assess the potential involvement of estrogen receptors (ER) on the effects of agathisflavone following LPC treatment, slices were pre-incubated for 2 h in medium containing the selective ER-α antagonist MPP dihydrochloride at 10 nM (1,3-Bis(4-hydroxyphenyl)-4-methyl-5-[4-(2-piperidinylethoxy)phenol]-1H-pyrazole dihydrochloride; Sigma), or the selective ER-β antagonist PHTPP at 1 μM (4-[2-Phenyl-5,7-bis(trifluoromethyl) pyrazolo[1,5-*a*]pyrimidin-3-yl]phenol; Tocris). Finally, slices were incubated for a further 2 DIV in medium containing 10 μM agathisflavone supplemented either with 10 nM MPP dihydrochloride, 1 μM PHTPP or 0.1% DMSO vehicle (LPC + DMSO condition). After 10 DIV, slices were either processed for RT-qPCR or fixed in 4% Paraformaldehyde (PFA) for immunohistochemistry (see below).

### 2.4 Immunohistochemistry

Prior to processing for immunohistochemical labelling, slices were washed with 0.1 M Phosphate Buffer Saline (PBS) then fixed with 4% PFA for 1 h, followed by further washes in PBS and either processed for immunostaining or stored at 4 °C in a solution of 0.05% Sodium Azide in PBS until ready for use. Slices were then washed in PBS and incubated overnight in 1% Triton X-100 in PBS at 4 °C, followed by a blocking step using 20% bovine serum albumin (BSA) in 0.1% Triton in PBS for 3 h, after which slices were incubated overnight with primary antibodies diluted in a solution of 1% normal goat serum (NGS) and 1% Triton-X in PBS. Oligodendroglial lineage cells and myelin were identified using rat anti-myelin basic protein (MBP) (1:300, Millipore, MAB386), mouse anti-APC/CC-1 (1:400, Calbiochem, OP80), rabbit anti-Chondroitin sulfate proteoglycan (NG2) (1:500, Millipore, MAB5384); neurons and axons were identified by using mouse anti-Neurofilament 70 kDa (NF70) (1:300, Millipore, MAB1615), mouse anti-calbindin D-28k (1:1000, Swant, 300PUR); microglia were immunostained with rabbit anti-Iba1 (1:1000, WAKO, 019-19741), M1-phenotype rat anti-CD16/32 (1:400, BD Pharmingen, 553142), M2-phenotype goat anti-CD206 (1:400, R&D Systems, AF2535); proliferating cells were identified by mouse anti-Ki67 (1:300, BD Pharmingen, 550609) and apoptotic cells by rabbit anti-cleaved Caspase-3 antibody (Asp175, 1:300, Cell Signalling, 9661S). Following overnight incubation in primary antibodies, slices were washed three times in 0.1% Triton-X in PBS prior to incubation for 3 h with the appropriate secondary antibodies (Alexa-fluor 568, 405, 488, 647, 1:500, Invitrogen) and the nuclear dye Hoechst33342 (1:500, Fisher, 11544876). Slices were then washed three times and mounted with Fluoromont-G (Invitrogen). Images were acquired using confocal microscopy (Zeiss LSM 710).

### 2.5 Cell quantification and myelin/axons index

Photomicrographs were obtained using a laser scanning confocal microscope (Zeiss LSM710) and 10 z-stacks of 1.0 μm each were acquired using a 20X objective. Cell counts of Sox10-EGFP+, and Iba-1+ cells were performed in a constant field of view (FOV, 708.49 × 708.49 x 10 μm) on images from white matter, whereas NG2+ cells were counted in the molecular layer. For MBP or neurofilament (NF) quantification, grids of 30 μm^2^ were used to quantify the myelin and axonal index by counting the number of intersections between MBP + and NF + fibres on a grid, and the extent of myelination was expressed as a percentage of the number of MBP^+^/NF^+^ axons over the total NF + axons.

### 2.6 Microglial analysis

Microglial morphological analysis was performed as previously described [24]. Confocal photomicrographs of Iba1+ cells were obtained in 4 *z*-stacks of 2 μm each acquired using a 63X objective. Cross-sectional area of microglial somata was measured in 20 cells per image (FOV, 708.49 x 708.49 μm). Binary and skeleton reconstructions of the z-stacks of confocal images were obtained using ImageJ-Win64 and AnalyzeSkeleton (2D/3D) plugin [25], adjusting brightness, unsharp mask, and despeckle to ensure process visualization prior to the conversion to binary and skeletonized images. The data from each image (summed number of endpoints and summed process length) was divided by the number of microglia in the image (20 microglial cells per each image); data was presented as a individual values column graphs to better illustrate the full range and patterns of the parameters measured. Quantification of microglia-oligodendrocyte contacts was adapted from the method of Barcia et al. [26], whereby the number of Iba1+ microglia cell bodies contacting Sox10-EGFP + oligodendrocyte cell bodies (B-B) and Iba1+ processes contacting Sox10-EGFP + oligodendrocyte cell bodies (Pr-B) were counted per constant FOV.

### 2.7 Quantitative polymerase chain reaction (RT-qPCR)

Quantitative real-time PCR (RT-qPCR) was performed using PrecisionPLUS qPCR Master Mix. Slices were removed from the insert, kept on RNA later and stored at −80 °C until ready to be processes for RNA extraction. Total RNA was isolated from cerebellar slices with QIAzol® Lysis Reagent according to the manufacturer's specifications. Total RNA was purified from cerebellar slices using RNeasy Plus Micro Kit (Qiagen, Hilden, Germany). Concentration and purity of RNA were determined by spectrophotometric analysis using a spectrophotometer (NanoDrop, ND-1000). For cDNA synthesis, the RNA was reverse transcribed into first-strand cDNA (NanoScript 2RT kit, Primerdesign, Southampton, UK) prior to RT-qPCR analysis. Custom designed RT-qPCR primers (Primerdesign, Southampton, UK), housekeeping genes and a PrecisionPLUS qPCR Master Mix (Primerdesign, Southampton, UK) were used in a 20-μL reaction. Thermocycling conditions were applied on LightCycler® Roche 96 and performed according to manufacturer's specifications (enzyme activation for 2 min, at 95 °C; denaturation for 10 s, at 95 °C, data collection for 60 s at 60 °C). Fluorogenic data was collected through the SYBR**®green** channel. The assays corresponding to the genes quantified in this study were: Ifng (ID 15978), Tnf (ID 21926), Il1b (ID 16176), Il6 (ID 16193), Il18 (ID 16173), Nos2 (ID 18126), Cx3cr1 (ID 13051), Cxcl10 (ID 15945), Trem2 (ID 83433), Inhba (ID 16323), C1qa (ID 12259), Nlrc4 (ID 268973), Nlrp3 (ID 216799), Arg1 (ID 11846), Il10 (ID 16153), Tgfb1 (ID 21803), Cntf (ID 12803), Egfr (ID 13649) and Gabrb1 (ID 14400). The actin beta (Actb, ID 11461) and Hypoxanthine Phosphoribosyl Transferase 1 (Hprt1, ID 15452) targets were used as reference genes (endogenous controls) for normalization of gene expression data. Data were analyzed using the 2–ΔΔCt method. Results represent the average of 3 independent experiments.

### 2.8 Molecular docking

Molecular docking analysis was performed using DOCK 6.8 [27], with the accessory programs DOCK 6.8 (DMS, SPHGEN, and SPHERE_SELECTOR) for search space delimitation [28,29] and the molecular properties were calculated by the GRID program in its default configuration using the Grid Score function (force field-based function) [30]. 3D structures of the proteins (PDB: 1 FCX; 4ZSH; 5KCF and 1YYE) were obtained from the macromolecular structures bank Protein Data Bank [31] and prepared through the DockPrep module in the Chimera program 1.10.1 [32]; water molecules and crystallization artefacts were removed and addition of the polar hydrogen atoms and charges (AM1-BCC) was performed. Evaluation of the scoring function was performed by the root-mean-square-deviation (RMSD) value between the conformation of the best pose calculated pose after docking and the crystallographic pose of that ligand. The interactions of the agathisflavone molecule with the RAR, RXRα, RXRγ and α and β estrogen receptors were analyzed with the aid of the PLIP program.

### 2.9 Statistical analyses

Statistical analysis was performed using GraphPad Prism 5. We first analyzed the data regarding their normality and tested if they had a Gaussian distribution [33]. For data with a normal distribution, we performed one- or two-way analysis of variance (ANOVA), as appropriate, followed by Bonferroni´s post-hoc test, or paired t-tests were used to compare the difference between two treatments, when applicable; normally distributed data are expressed as mean + SEM. For samples with a non-Gaussian distribution, we used non-parametric tests, Kruskal-Wallis followed by Dunn’s multiple comparison test; non-parametric data were expressed as medium + interquartile range (IQR), which is appropriate to indicate variability/dispersion among non-normal samples [33]. Confidence intervals were defined at a 95% confidence level (*p* < 0.05 was statistically significant).

## 3 Results

### 3.1 Agathisflavone enhances remyelination and induces oligodendrocyte proliferation in organotypic cerebellar slices

**Fig. 1:**
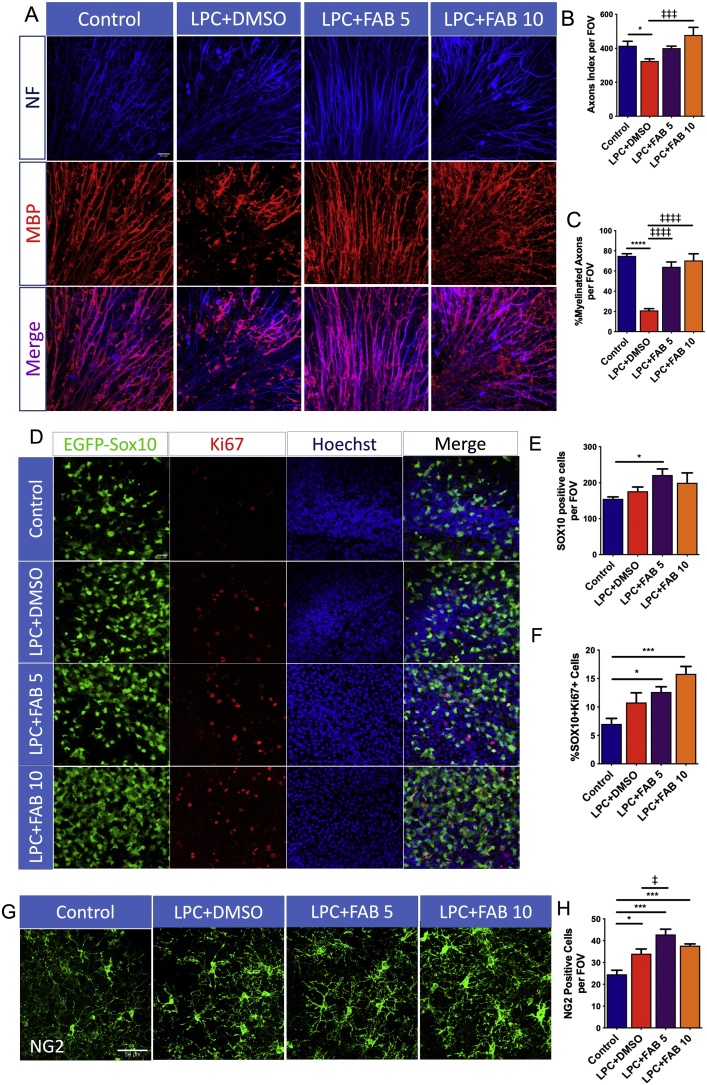
**Agathisflavone enhances remyelination and induces oligodendrocyte proliferation in organotypic cerebellar slices culture.** Organotypic cerebellar slices from P10-12 Sox10-EGFP mice were maintained for 7 DIV and then treated with LPC for 15–17 h, followed by agathisflavone (FAB) at 5 or 10 μM for a further 2 DIV, or 0.1% DMSO vehicle. **(A)** Photomicrographs showing the cerebellar white matter stained with MBP (red) and NF (blue); scale bar 20 μm. **(B, C)** Bar graphs showing the NF + axon index (C) and the percentage of MBP+/NF + myelinated axons (D) per constant field of view (FOV). (**D)** Oligodendrocyte lineage Sox10-EGFP + cells (green), immunostained for the proliferating marker Ki67 (red) and counterstained with Hoechst nuclear dye (blue); scale bar 20 μm. **(E, F)** Bar graphs showing the number of Sox10+ cells per FOV (E) and the percentage of SOX10+/Ki67+ cells (F) in a constant FOV. **(G)** Photomicrographs of OPCs immunolabelled for NG2; scale bar 50 μm. **(H)** Bar graph showing the number of NG2 + OPCs per FOV. Data are expressed as the mean ± SEM (n = 6); *p < 0.05, *** *p*<0.001, ****p < 0.0001 (comparing controls to treatment groups); ‡*p* < 0.05, ‡‡‡*p* < 0.001, ‡‡‡‡*p* < 0.0001 (comparing LPC+DMSO to LPC+FAB5 and LPC+FAB10), One-way ANOVA followed by Tukey’s post-hoc test. (For interpretation of the references to colour in this figure legend, the reader is referred to the web version of this article.)

The effect of agathisflavone in response to a demyelinating insult was examined in *ex vivo* cerebellar slices that were treated with LPC [19]. Cerebellar slices from P10-12 mice were kept in normal medium for 7 DIV to allow normal myelination to occur before exposure to LPC for 15–17 h and subsequent treatment either with agathisflavone (5 and 10μM) or 0.1% DMSO vehicle for a 2DIV, after which slices were examined for the extent of myelination, using immunolabelling for MBP and NF (Fig. 1). The effects of LPC were first compared to untreated slices that were maintained in normal medium for 7 DIV and the results show that LPC treatment resulted in a minor, but statistically significant, decrease in the axon index (Fig. 1A, B), and a marked 4-fold decrease in the proportion of MBP+/NF + myelinated axons (Fig. 1A, C). The effect of LPC was clearly counteracted by treatment with agathisflavone at both concentrations tested (Fig. 1A–C).

Proliferation of OPCs is important for regenerating oligodendrocytes following demyelination [3], hence we examined the effects of agathisflavone in cerebellar slices from Sox10-EGFP mice to identify all oligodendrocyte lineage cells and immunostaining for the cell proliferation marker Ki67 to identify actively proliferating OPCs (Fig. 1D). Exposure to LPC did not significantly alter the total number of Sox10+ oligodendrocyte lineage cells (Fig. 1E) or induce their proliferation (Fig. 1F), compared to untreated controls. In contrast, Sox10+ cells were significantly increased after treatment with 5 μM agathisflavone and Ki67^+^/Sox10^+^ cells were significantly increased at both 5 and 10 μM agathisflavone, compared to untreated controls (Fig. 1D–F). To further investigate the effect of agathisflavone specifically on OPCs, the proliferating cells of the oligodendrocyte lineage, we performed NG2 immunostaining (Fig. 1G). Notably, NG2 + OPC were increased following LPC treatment, compared to controls (Fig. 1H), consistent with the early stage of spontaneous repair that occurs in this model [19], and this was significantly increased further by agathisflavone at 5 μM (Fig. 1H).

### 3.2 Agathisflavone increases mature oligodendrocytes number and prevents oligodendrocyte apoptosis

**Fig. 2:**
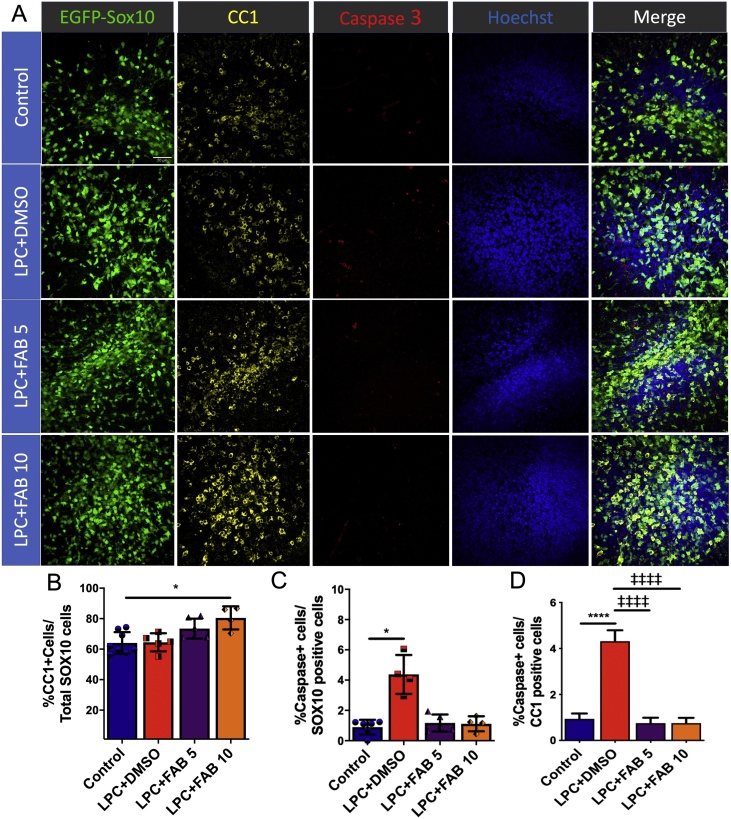
**Agathisflavone increases mature oligodendrocyte cells number and prevents oligodendrocyte apoptosis.** Organotypic cerebellar slices from P10-12 Sox10-EGFP mice were maintained for 7 DIV and then treated with LPC for 15–17 h, followed by agathisflavone (FAB) at 5 or 10 μM for a further 2 DIV, or 0.1% DMSO vehicle. **(A)** Oligodendrocyte lineage cells identified by expression of the Sox10-EGFP reporter (green), immunolabelling for CC1 for mature oligodendrocytes (yellow) and active Caspase 3 for apoptotic cells (red), and counterstained with Hoechst nuclear dye (blue); scale bar 50 μm. **(B, C)** Individual values column graphs showing the percentage of CC1+ /Sox10+ cells (B), Caspase+/SOX10+ cells (C) and Caspase+/CC1+ cells (D) in a constant FOV; data are expressed as mean ± SEM (n = 4–6); *p < 0.05, ****p < 0.0001 (comparing controls to treatment groups); ‡‡‡‡*p* < 0.0001 (comparing LPC+DMSO to LPC+FAB5 and LPC+FAB10), One-way ANOVA followed by Tukey’s post-hoc test. (For interpretation of the references to colour in this figure legend, the reader is referred to the web version of this article.)

Following proliferation, the differentiation and survival of OPCs is essential for remyelination to occur [3]. We examined the protective effect of FAB on oligodendrocytes in cerebellar slices from Sox10-EGFP mice immunolabelled for CC1, a marker for mature oligodendrocytes, and cleaved caspase-3, a classical marker for apoptosis (Fig. 2A). The results demonstrate that agathisflavone increased the proportion of CC1+/Sox10+ mature oligodendrocytes and this was statistically significant at 10 μM but not at 5 μM (Fig. 2B), compared to controls. There was a significant increase in apoptosis-mediated cell death following LPC treatment in Sox10+ and CC1+ oligodendrocytes (Fig. 2C, D), an effect that was abrogated by agathisflavone at both 5 and 10 μM (Fig. 2C, D).

### 3.3 Agathisflavone modifies microglial activation and modulates interactions with oligodendrocytes

**Fig. 3:**
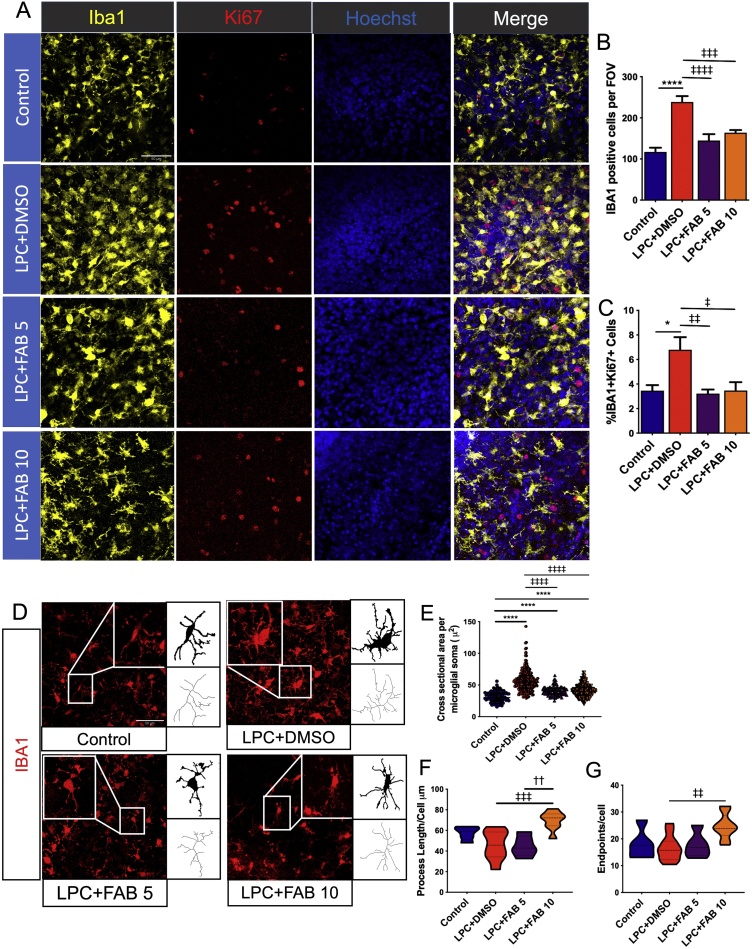
**Agathisflavone modifies microglial activation state.** Organotypic cerebellar slices from P10-12 Sox10-EGFP mice were maintained for 7 DIV and then treated with LPC for 15–17 h, followed by agathisflavone (FAB) at 5 or 10 μM for a further 2 DIV, or 0.1% DMSO vehicle. **(A)** Microglial proliferation was analyzed by immunolabelling for IBA1 (yellow) and Ki67 (red), counterstained with the nuclear dye Hoechst (blue). **(B, C)** Bar graph showing the number of IBA1+ microglia (B) and the percentage of IBA1+/Ki67+ proliferating microglia (C); data are expressed as the mean ± SEM (n = 5–11) and tested for significance using One-way ANOVA followed by Tukey’s post-hoc test. **(D)** Photomicrographs and binary and skeletonized IBA + microglia illustrating morphological differences in the different treatment groups; scale bar 50 μm. **(E, F, G)** Individual values violin plots of microglial soma size per microglial cell (20 microglial cells/image were analyzed) (E) and violin graphs of process endpoints (F) and length (G) per microglial cell; data are expressed as the median ± IQR; *p < 0.05, ****p < 0.0001 (comparing controls to treatment groups); ‡*p* < 0.05, ‡‡*p* < 0.01, ‡‡‡*p* < 0.001, ‡‡‡‡*p* < 0.0001 (comparing LPC+DMSO to LPC+FAB5 and LPC+FAB10); ††*p* < 0.01 (comparing LPC+FAB5 to LPC+ FAB10); Kruskal-Wallis test followed by Dunns. (For interpretation of the references to colour in this figure legend, the reader is referred to the web version of this article.)

Microgliosis plays an important role in the inflammatory response and is an integral part of the remyelination process following injury [34]. In order to investigate the effect of agathisflavone as a modulator of microglial activation, we immunostained for the microglial marker IBA1 and the proliferative marker Ki67 (Fig. 3A). The number of IBA1+ cells (Fig. 3B) and Iba1+/Ki67+ cells (Fig. 3C) were significantly increased after LPC treatment, compared to controls, and this effect was completely abolished by treatment with agathisflavone at both 5 and 10 μM. The results are consistent with agathisflavone having a dampening effect on LPC-induced microglial activation. We examined this further by using morphological reconstructions of IBA1+ microglial changes that are characteristic of their activation status (Fig. 3D). In controls, microglia had small somata and ramified processes typical of quiescent or non-activated cells (Fig. 3D, E), whilst in LPC-treated slices microglial somata were markedly increased compared to control (Fig. 3D, E), suggesting microglial activation [35]; process number (Fig. 3F) and length (Fig. 3G) were not altered in LPC. Treatment with agathisflavone (5 μM and 10 μM) resulted in a significant reduction in microglial soma size compared to LPC (Fig. 3D, E), and 10 μM agathisflavone increased microglial process number and length (Fig. 3F, G).

**Fig. 4:**
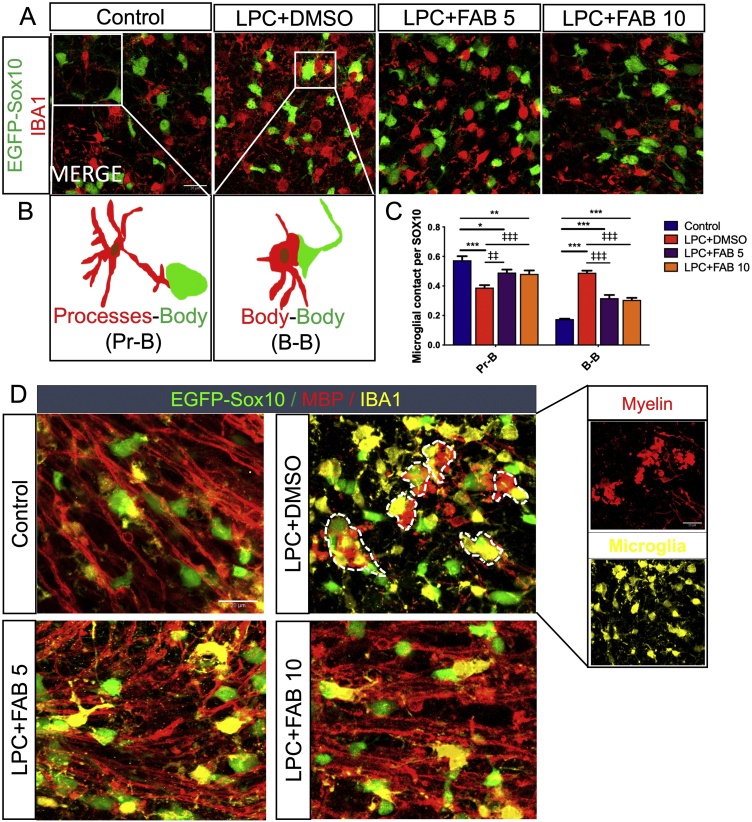
**Agathisflavone modulates microglia-oligodendrocyte interactions.** Organotypic cerebellar slices from P10-12 Sox10-EGFP mice were maintained for 7 DIV and then treated with LPC for 15–17 h, followed by agathisflavone (FAB) at 5 or 10 μM for a further 2 DIV, or 0.1% DMSO vehicle. **(A)** Photomicrographs of IBA1 immunostaining (red) and SOX10-EGFP+ oligodendrocytes (green) showing oligodendrocytes-microglia contacts in the different treatment groups; scale bar 20 μm. **(B)** Diagram illustrating microglial processes contacting oligodendrocytes body (Pr-B), or apposition of microglial and oligodendrocyte cell bodies (B—B). **(C)** Grouped bar graph showing the number of microglial contacts per SOX10+ cells; data are expressed as the mean ± SEM (n = 6), *p < 0.05, **p < 0.01, ***p < 0.001 (comparing control to treatment groups); ‡‡*p* < 0.01, ‡‡‡*p* < 0.001 (comparing LPC-DMSO to LPC+FAB5 and LPC+FAB10; two-way ANOVA followed by Tukey’s post-hoc test. **(D)** Photomicrographs of slices illustrating Sox10-RGFP + oligodendrocytes (green) and immunolabelling for MBP (red) and Iba1 (yellow), showing interrelationships between microglia, oligodendrocytes, and myelinated fibres in the different treatment groups; clusters of IBA1+ microglia around myelin debris and oligodendrocytes are evident following LPCv treatment and are rarely observed in controls or following agathisflavone treatment. (For interpretation of the references to colour in this figure legend, the reader is referred to the web version of this article.)

In addition, we quantified the number of contacts between IBA1^+^ microglial cells and Sox10-EGFP + oligodendrocytes (Fig. 4A, B), to understand if agathisflavone regulates microglia-oligodendrocyte interactions, which is an indication of their capacity for oligodendrocyte elimination [36]. In LPC-treated slices, we observed a significant reduction of microglia-oligodendrocyte contacts via processes (Pr-B, Fig. 4C) and an increase in their contact via cell body (B-B), and these effects were reversed by agathisflavone treatment (Fig. 4C). Microglia-oligodendrocyte interactions were further investigated by double immunostaining for IBA1 and MBP in Sox10-EGFP cerebellar slices (Fig. 4D). The results show that in controls, microglia interact closely with oligodendrocytes and myelin, whereas after LPC damage, microglial clusters were observed surrounding myelin debris and Sox10-EGFP^+^ oligodendrocytes. Treatment with agathisflavone at both concentrations enhanced remyelination and reversed the microglia-cluster formation with oligodendrocytes, an effect consistent with agathisflavone restoring normal microglia-oligodendrocyte interactions and tissue homeostasis.

### 3.4 Agathisflavone alters microglial activation state

**Fig. 5:**
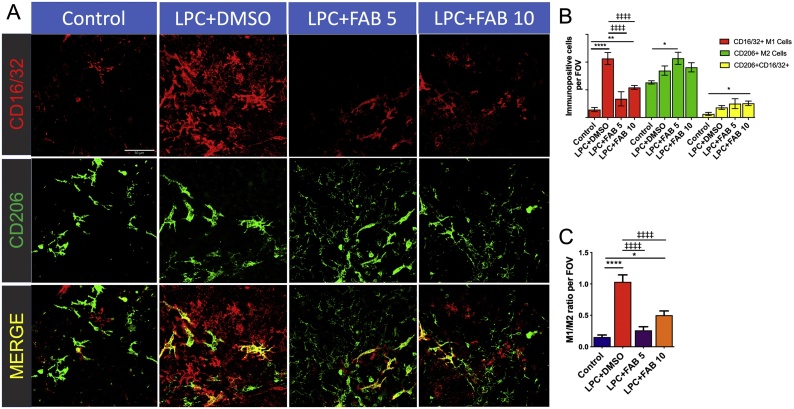
**Agathisflavone promotes a microglial polarization from a M1 to a M2 profile.** Organotypic cerebellar slices from P10-12 mice were maintained for 7 DIV and then treated with LPC for 15–17 h, followed by agathisflavone (FAB) at 5 or 10 μM for a further 2 DIV, or 0.1% DMSO vehicle. **(A)** Microglial profile analyzed by double immunofluoresecence labelling for the M1 pro-inflammatory marker CD16/32 (red) and M2 anti-inflammatory marker CD206 (green), where co-expression appears yellow; scale bar 50 μm. **(B, C)** Bar graphs showing the number of CD16/32+, CD206+ and CD206+/CD16/32+ cells (B) and the M1/M2 ratio (C); data are expressed as the mean ± SEM (n = 6), *p < 0.05, **p < 0.01, ****p < 0.0001 (comparing control to treatment groups); ‡‡‡‡*p* < 0.0001 (comparing LPC-DMSO to LPC+FAB5 and LPC+FAB10); One-way ANOVA followed by Tukey’s post-hoc test. (For interpretation of the references to colour in this figure legend, the reader is referred to the web version of this article.)

**Fig. 6:**
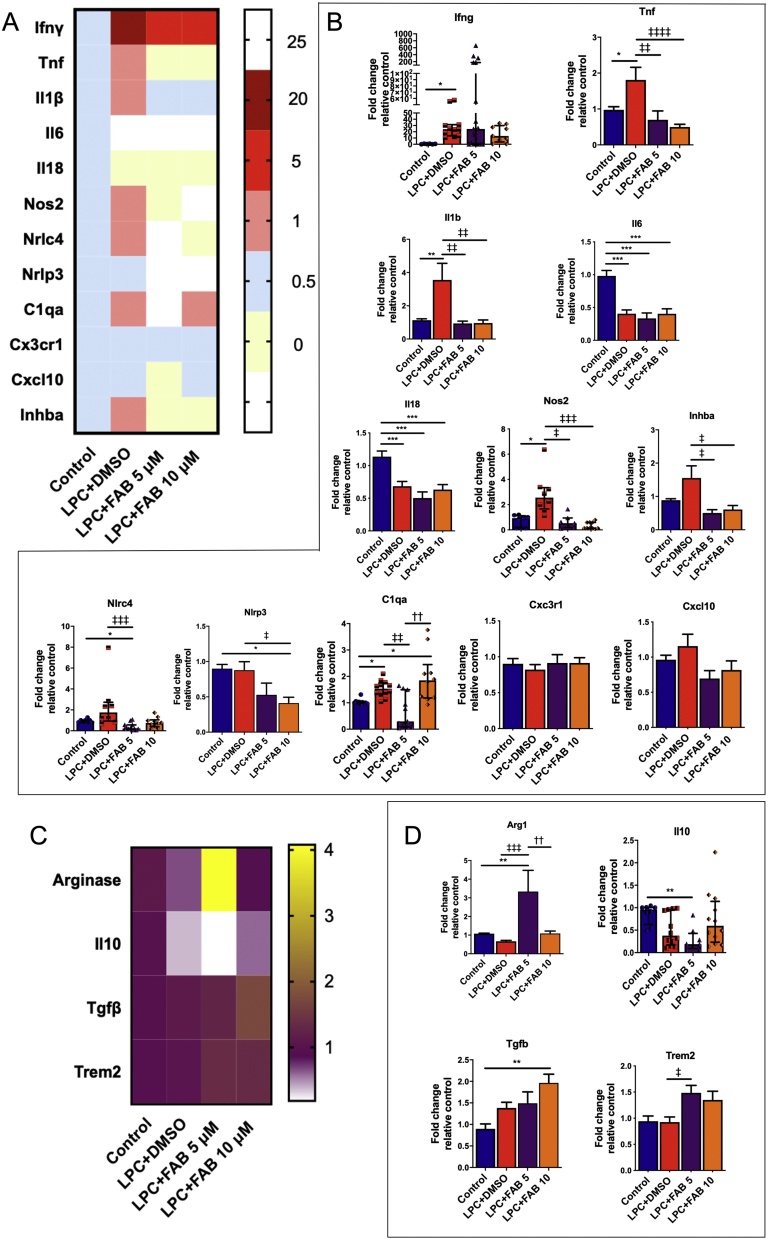
**Agathisflavone modulates transcript levels of neuroinflammatory genes.** Organotypic cerebellar slices from P10-12 mice were maintained for 7 DIV and then treated with LPC for 15–17 h, followed by agathisflavone (FAB) at 5 or 10 μM for a further 2 DIV, or 0.1% DMSO vehicle. (**A, B)** Heat map showing the expression of neuroinflammatory genes (A) and respective graphs (B) of RT-qPCR analysis showing the expression of neuroinflammatory genes. **(C, D)** Heat map showing the expression of regulatory factors (C) and respective graphs (D). Data are expressed as the mean ± SEM or median ± IQR (n = 4); *p < 0.05, **p < 0.01, ***p < 0.001 (comparing control to treatment groups); ‡*p* < 0.05, ‡‡*p* < 0.01, ‡‡‡*p* < 0.001, ‡‡‡‡*p* < 0.001(comparing LPC-DMSO to LPC+FAB5 and LPC+FAB10); ††*p* < 0.01 (comparing LPC+FAB5 to LPC+FAB10); samples with Gaussian distribution (bar graphs) were analyzed by one-way ANOVA followed by Tukey’s post-hoc test, non-parametric samples (individual values column graphs) by Kruskal-Wallis followed by Dunns.

Results presented above indicate that agathisflavone modulates microglial activation. We examined this further by assessing the microglial profile using immunostaining (Fig. 5) and RT-qPCR (Fig. 6). Microglia undergo activation in response to pathology and numerous studies have characterised microglial activation as being pro-inflammatory M1 or anti-inflammatory M2, with a number of key transcriptional regulators that serve as central switches to regulate M1 and M2 genes [37]. This polarization of M1/M2 phenotypes is an oversimplification [5], but it has been reported that a switch from an ‘M1’ to an ‘M2’ profile reflects a change from demyelination to remyelination and repair [6,7]. Hence, we examined whether agathisflavone can regulate microglia phenotype using double immunofluorescence labelling for CD16/32 (CD16, Fc gamma III Receptor; CD32, Fc gamma II Receptor) and CD206 (a pattern recognition receptor), respectively considered ‘M1’ pro-inflammatory and ‘M2’ anti-inflammatory profile markers (Fig. 5A). The immunofluorescence analysis demonstrates that LPC increased the number of CD16/32^+^ M1-like microglia, compared to controls, and this was significantly reduced by agathisflavone at both concentrations (Fig. 5B); moreover at 5 μM, agathisflavone significantly increased the number of CD206+ M2-like microglia, while 10 μM agathisflavone significantly increased CD16/32^+^ CD206^+^ microglia (Fig. 5B). Recognizing that the polarization of M1 and M2 phenotypes is an over-simplification, our data demonstrate that the ‘M1/M2’ ratio is markedly altered in LPC treatment and this was completely reversed by agathisflavone at both concentration (Fig. 5C), consistent with evidence that the ‘M1’ phenotype is associated with inflammation and demyelination, whereas the ‘M2’ phenotype supports remyelination and repair [6,7].

To confirm the effect of agathisflavone on neuroinflammation, we investigated the effect of the treatments on neuroinflammatory-produced genes, which are mainly, but not exclusively secreted by microglia, through RT-qPCR (Fig. 6). We observed that LPC upregulated mRNA expression of the classical inflammatory genes IFNγ, TNFα, IL1β, NOS2 and C1QA, and of the activin A (INHBA) gene, (Fig. 6A, B) and downregulated IL6 and IL18 (Fig. 6A, B), an effect that might be due to a transient delay in the expression of these proinflammatory cytokines within the timepoint analyzed. In contrast, agathisflavone at both concentrations, controls mRNA expression of inflammatory (TNF, IL1β, NOS2, and C1QA), regulatory (Arginase, TGFB and TREM2, Fig. 6C, D), and INHBA, which is present at the site of demyelinated lesions and regulates myelination [38]. Moreover, in comparison to control and LPC treated cultures, agathisflavone at both concentrations reduced the mRNA expression of NLRC4 and NLRC3 inflammasome genes.

### 3.5 Agathisflavone regulates reactive astrogliosis and protects neurons

**Fig. 7:**
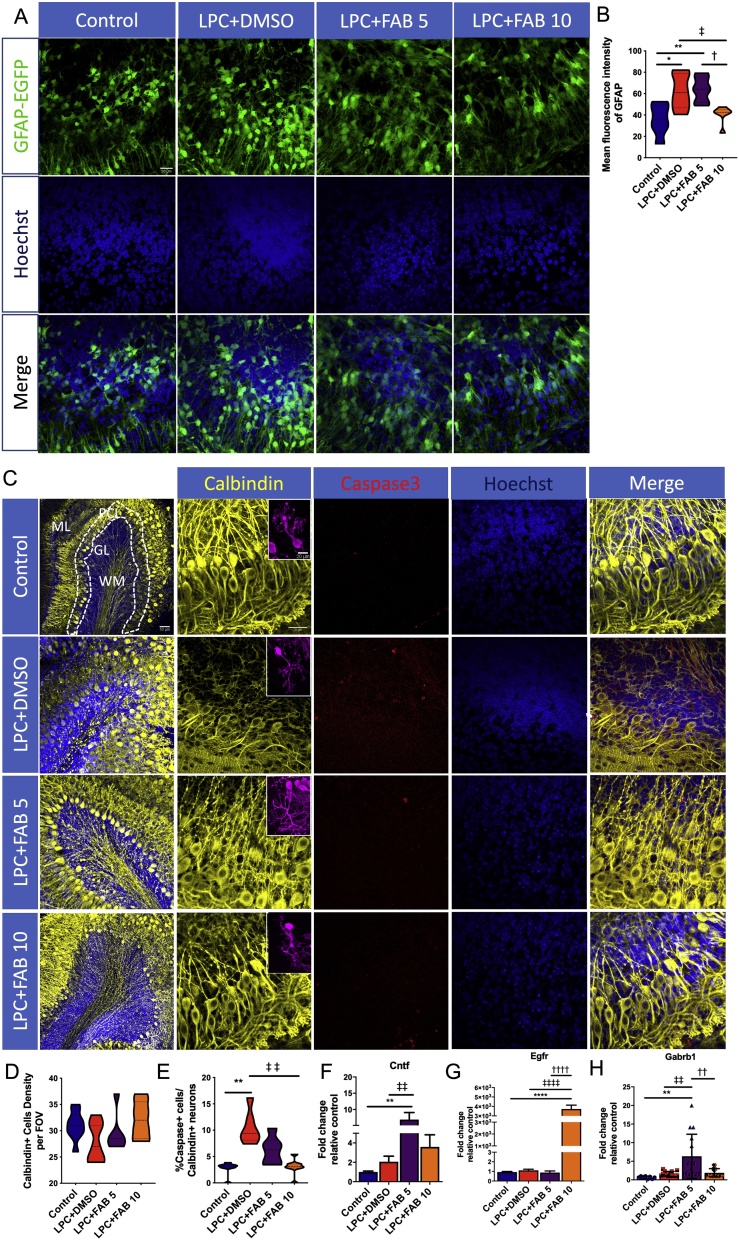
**Agathisflavone regulates reactive astrogliosis and is neuroprotective.** Organotypic cerebellar slices from P10-12 mice were maintained for 7 DIV and then treated with LPC for 15–17 h, followed by agathisflavone (FAB) at 5 or 10 μM for a further 2 DIV, or 0.1% DMSO vehicle. . **(A)** Photomicrographs illustrating GFAP-EGFP + astrocytes (green) and Hoescht stained nuclei (blue); scale bar 20 μm. **(B)** Violin graphs showing the mean fluorescence intensity of GFAP in the different treatment groups. **(C)** Photomicrographs of Purkinje neurons immunolabelled for Calbindin (yellow) and the apoptosis marker cleaved Caspase-3 (red) and counterstained with Hoechst (blue). The panels on the left side show entire cerebellar lobules and the organization of its layers (ML: Molecular layer; PCL: Purkinje cells layer; GL: Granular layer; WM: **White** matter); scale bar 50 μm. The remaining panels focus on the PCL; scale bar 50 μm. Insets illustrate individual Purkinje cells ; scale bar 20 μm. **(D, E)** Violin graphs showing the number of Calbindin + cells per FOV (D) and the percentage of Caspase+ /Calbindin + cells (E). **(F, G, H)** RT-qPCR analysis Cntf (F), Egfr (G) and Gabbr1 mRNA expression in cerebellar slices in the different treatment groups; data are expressed as the mean ± SEM or median ± IQR (n = 5); *p < 0.05, **p < 0.01, ****p < 0.0001 (comparing control to treatment groups); ‡*p* < 0.05, ‡‡*p* < 0.01, ‡‡‡‡*p* < 0.001 (comparing LPC-DMSO to LPC+FAB5 and LPC+FAB10); †*p* < 0.05, ††*p* < 0.01 and ††††*p* < 0.0001 (comparing LPC+FAB5 to LPC+FAB10); samples with Gaussian distribution (bar graphs) were analyzed by One-way ANOVA followed by Tukey’s post-hoc test, non-parametric samples (individual values column graphs) by Kruskal-Wallis followed by Dunns. (For interpretation of the references to colour in this figure legend, the reader is referred to the web version of this article.)

Reactive astrogliosis is important in CNS inflammation and neuronal damage [39]. The effects of agathisflavone on astrocytes were examined in cerebellar slices from GFAP-EGFP mice (Fig. 7A). In comparison with the control, LPC increased GFAP fluorescence intensity, an indicator of reactive astrogliosis, and this was significantly decreased by 10 μM agathisflavone, but not at 5 μM (Fig. 7A, B). The effect of agathisflavone on neurons was examined using immunostaining for Calbindin, a marker for Purkinje neurons [40], and co-stained with cleaved caspase-3 for cell death (Fig. 7C). The overall number of Purkinje neurons was unaltered by LPC and by treatment with agathisflavone (Fig. 7D), but the number of Calbindin+/Caspase3+ neurons was increased in LPC and this was reversed by agathisflavone treatment, indicating that agathisflavone is neuroprotective (Fig. 7E). To further understand the mechanism by which agathisflavone mediates neuronal protection, we WE conducted RT-qPCR to measure mRNA expression of the neurotrophic factors ciliary neurotrophic factor (Cntf,), epidermal growth factor receptor (Egfr), and neuronal GABA b1 receptor subunit (Gabrb1). Treatment with agathisflavone significantly increased expression of Cntf (Fig. 7F), Egfr (Fig. 7G) and Gabrb1 (Fig. 7H), compared to controls and LPC treated slices, with Cntf and Gabrb1 being more significantly affected by 5μM agathisflavone and Egfr most markedly increased by 10μM agathisflavone.

### 3.6 Estrogen receptor (ER) activation is required for agathisflavone to inhibit microgliosis and promote remyelination

**Fig. 8:**
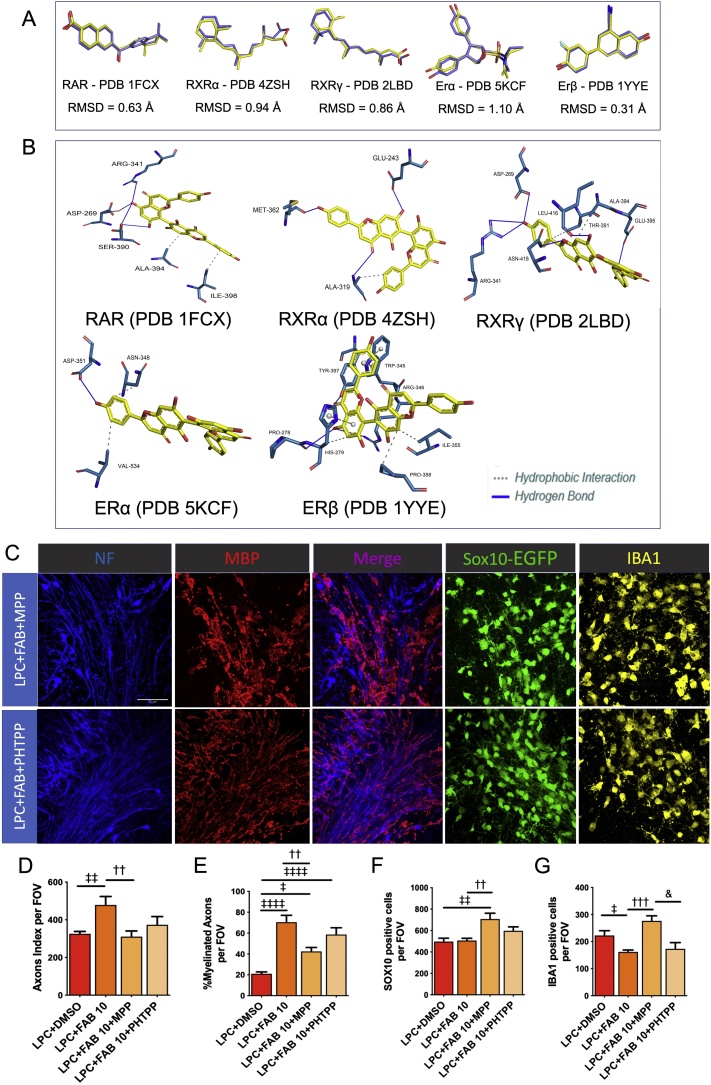
**Estrogen receptor (ER) activation is required for agathisflavone to inhibit microgliosis and promote remyelination. (A)** Root mean square deviation (RMSD) values and rod representation of crystallographic ligand pose (lilac) and the best pose of this ligand generated by DOCK 6.8 (yellow) for each complex. Distances less than 2 Å between the calculated pose and the crystallographic pose indicates that the program was successful in reproducing the experimental data **(B)** Representation of interactions between agathisflavone (FAB) and retinoic and estrogen receptors; the captions are described in the figure. **(C–G)** Organotypic cerebellar slices from SOX10-EGFP animals were maintained for 7DIV, then exposed to LPC for 15–17 h, followed by 2 h pretreatment with the selective ER-α antagonist MPP dihydrochloride at 10 nM (1,3-Bis(4-hydroxyphenyl)-4-methyl-5-[4-(2-piperidinylethoxy)phenol]-1H-pyrazole-dihydrochloride), or the selective ER-β antagonist PHTPP at 1 μM (4-[2-Phenyl-5,7-bis(trifluoromethyl) pyrazolo[1,5-*a*]pyrimidin-3-yl]phenol), which were kept together with 10 μM FAB for a further 2 DIV. (C) Oligodendrocytes were identified by the Sox10-EGFP reporter (green) and slices were immunolabeled for MBP (red), neurofilament (blue) and Iba-1 (yellow); scale bar: 20 μm. Bar graphs compare LPC and LPC + FAB 10 μM with the effects of the ER antagonists MPP and PHTPP on the NF + axon index (D), the percentage of MBP+/NF + myelinated axons (E), the number of Sox10-EGFP + oligodendrocytes (F) and the number of Iba1+ microglia (G); data are expressed as the mean ± SEM (n = 5); ‡*p* < 0.05, ‡‡*p* < 0.01, ‡‡‡‡*p* < 0.0001 (comparing LPC-DMSO to other treatment groups); ††*p* < 0.01 and †††*p*<0.001 (comparing LPC + FAB10 to LPC + FAB10+MPP); &*p* < 0.05 (comparing LPC+FAB10+MPP to LPC+FAB10+PHTPP); One-way ANOVA followed by Tukey’s post-hoc test. (For interpretation of the references to colour in this figure legend, the reader is referred to the web version of this article.)

In order to understand how molecular recognition occurs between agathisflavone and molecular targets, we performed molecular docking, a powerful tool that is widely used in drug design, to identify complementarity between molecules and their potential targets [41]. Previous studies have indicated ER interact with other nuclear receptors [42] and the neuroprotective actions of agathisflavone have been shown to involve ER and RAR [14]. Hence, we investigated the molecular affinity between agathisflavone and ERα, ERβ and RAR, together with retinoid X receptors (RXR)α and RXRγ (Fig. 8). The docking success refers to when a ligand crystallographic pose is close to a ligand calculated pose, which is obtained when the root-mean-square deviation (RMSD) is ≤2 Å. We demonstrated that all poses here calculated were ≤2 Å (Fig. 8A), completely validating the docking analysis. Superimposition with ligand binding sites identified molecular interactions of agathisflavone with the different receptors (Fig. 8B), and determination of the affinity energies demonstrated agathisflavone displays affinity with all the receptor targets (RAR -41.35 kcal/mol, RXRα -35.40 kcal/mol, RXRγ -36.29 kcal/mol, ERα -22.32 kcal/mol, ERβ -30.67 kcal/mol).

The chemical nature of the active site of the receptors and the presence of hydroxyl groups on agathisflavone favours the formation of hydrogen bonds, which is important for the molecular and selective recognition of ligands against the receptors [43]. As illustrated in Fig. 8B, in the agathisflavone -RAR complex, hydrogen bonds are formed with the amino acid Arg341, Asp269, Ser390, whilst hydrophobic interactions are formed with amino acids Ala394 and Ile398 of the RAR. In RXRα, agathisflavone forms hydrogen bonds with Glu-243, Met-362, Ala-319, together with hydrophobic interactions with the amino acid Ala-319 on the ligand binding site, whilst in the agathisflavone -RXRγ complex, hydrogen bonds form with amino acids Asp-269, Arg-341, Asn-418, Leu-416 and Glu-395 and hydrophobic interactions between the amino acids Thr-391 and Ala-394 and agathisflavone. The agathisflavone-ER complex displays formation of a hydrogen bond with Asp-351 of the ligand binding site of ERα, and hydrophobic interactions with the amino acid residues Asn-348 and Val-534, whereas in ERβ agathisflavone formed hydrogen bonds with amino acid residues Pro-278, Arg-346, Tyr-397 and Trp-345, hydrophobic interactions with Ile-355, Pro-358, His-279, and π-stacking with His-279 and Trp-345. Notably, hydrogen bond interactions in the phenol ring with Arg is involved in the biological activities of ER [44].

To investigate the involvement of ER signaling on microgliosis inhibition and remyelination induced by agathisflavone, antagonists of either the ERα (MPP) or ERβ (PHTPP) isoforms were added to cultures. Cerebellar slices were maintained in *ex vivo* organotypic culture for 7 DIV and exposed to the demyelinating agent LPC for 15–17 h, followed by incubation with MPP or PHTPP for 2 h, and then maintained in 10 μM agathisflavone for a further 2DIV (Fig. 8C). As above, the number of NF + axons and MBP+/NF + myelinated axons was significantly increased by agathisflavone treatment, compared to LPC, and this effect was partially ablated by blockade of ERα and, although blocking ERβ showed no statistical significance, the effect on remyelination was clearly altered (Fig. 8D, E). The number of SOX10-EGFP + oligodendrocytes was not altered in LPC or LPC + agathisflavone, but their number was significantly increased following blockade of ERα (Fig. 8F). In addition, agathisflavone significantly decreased microgliosis in response to LPC, and this effect was completely blocked by the ERα antagonist MPP, but not the ERβ antagonist PHTPP (Fig. 8G). Together these data suggest agathisflavone may interact with ERα to reduce microgliosis and enhance remyelination.

## 4 Discussion

This study demonstrates that the flavonoid agathisflavone promotes remyelination and regulates microglial activation in the lysolecithin model of demyelination in organotypic cerebellar slices. In this model, remyelination occurs in normal media after 4DIV, with marked remyelination appearing at 6DIV [19]. A key finding of the present study is that blockade of ER reduced the effects of agathisflavone on remyelination and microglia, and molecular docking analyses provided supporting evidence that agathisflavone may interact with ER. The results show that agathisflavone may represent a potential non-toxic therapy to promote repair in MS and other neuropathologies that involve myelin damage and neuroinflammation.

Lysolecithin (LPC)-mediated demyelination in cerebellar slices is a broadly used model for MS, because it mimics myelin damage and repair along a clearly defined time course [19]. Our results demonstrate that LPC induced oligodendroglial demise, as measured by Caspase-3 expression, together with prominent demyelination, as indicated by decreased MBP+/NF + axons. These effects of LPC were completely reversed by treatment with agathisflavone. The results demonstrate agathisflavone is protective for oligodendrocytes and promotes remyelination, as well as protecting axons against damage. Remyelination is dependent on proliferation and differentiation of OPCs [3], and our data demonstrated that agathisflavone increases proliferating Ki67+/NG2 + OPCs and increases the overall numbers of NG2 + OPCs, Sox10-EGFP^+^ cells and CC1^+^ mature oligodendrocytes. Overall, the results show that agathisflavone promotes OPC proliferation and differentiation, together with their survival, thereby stimulating remyelination following LPC treatment.

The CNS response against disease and injury involves complex interactions between microglia and the other cellular elements, oligodendrocytes, neurons, and astrocytes [4]. LPC treatment induced microglial activation and this was reversed by agathisflavone, which is a crucial event that helps drive remyelination [7]. Our data indicate that agathisflavone reduced LPC-induced microgliosis and microglial contacts with oligodendrocytes, as well as decreasing expression of important inflammatory molecules, such as Tnf, Il-1β, Nos2, C1q and Nlrc4, whilst augmenting expression of arginase, Tgf-β, and Trem2, which have critical roles in inflammatory control and oligodendrocyte differentiation [[45], [46], [47]]. Interestingly, agathisflavone did not reduce mRNA expression of IFNγ, which regulates microglia and is implicated in efficient remyelination [34]. Similarly, agathisflavone at 10 μM did not reduce C1q expression, which requires further investigation, since C1q is implicated in the activation of adult OPCs, a crucial step underlying myelin repair [48]. In addition, the pro-inflammatory cytokines IL6 and IL18 were not increased in LPC treated slices, consistent with previous evidence that LPC does not stimulate microglia to secrete IL6 in culture [49], although it remains possible that the mRNA’s measured could change transiently and be reduced again at the time-point analyzed. Additionally, agathisflavone reduces the expression of activin A (Inhba), which is expressed in microglia/macrophages present at the site of demyelinated lesions and supports myelin repair [38,57] []. Overall, our data indicated that agathisflavone altered the microglial activation state and promoted an anti-inflammatory phenotype. However, based on the mRNA data, we did not observe polarized M1 and M2 phenotypes following LPC and agathisflavone treatment, consistent with evidence that microglia are highly heterogeneous [50].

In cerebellar slices, LPC significantly increases GFAP-EGFP intensity, indicative of reactive astrogliosis, an effect that has been extensively observed and described in other studies [51]. Notably, reactive astrogliosis is significantly reduced by treatment with agathisflavone. The protective effects of agathisflavone on astrocytes will undoubtedly play a role in the observed effects on oligodendrogenesis and remyelination. Notably, astrocytes are a source of CNTF and EGF, and both Cntf and Egfr are increased by agathisflavone and have been shown to enhance oligodendrogenesis and accelerate remyelination [52,53]. Egfr also promote ppro migration of postnatal neural progenitors *in vitro* and *in vivo*. However, this marked upregulation of Egfr by agathisflavone needs further investigation [54]. Moreover, LPC significantly increases Caspase-3^+^ in Purkinje neurones and this is attenuated by agathisflavone, indicating its neuroprotective potential. Consistent with this, agathisflavone increases expression of Gabrb1, which are highly expressed in Purkinje neurones and downregulated in pathology [55]. The results demonstrate that agathisflavone is protective for neurones and modulates microgliosis and astrogliosis, which play important roles in tissue repair and remyelination.

A novel finding of our study is that ER activation is required for agathisflavone to inhibit microgliosis and promote remyelination. Notably, pharmacological inhibition indicated these effects are greatest through ERα receptors, although further studies are required before a role for ERβ receptors is excluded. In addition, our molecular docking analyses indicated that, in addition to ER, agathisflavone can interact with the nuclear receptors RAR and RXRα/γ. Our findings support evidence that ER interact via ligand-binding domains with RAR and RXR, thereby increasing their potential biological actions [42]. Additionally, previous work [14] in a model of glutamate-mediated neurotoxicity showed that agathisflavone exerted neurogenic effects via estrogen signalling and enhanced the neuroprotective properties of microglia and astrocytes. These findings provide a testable hypothesis by which agathisflavone regulates microglial activation, reactive astrogliosis, neuronal survival and myelination via interactions with multiple nuclear receptors, which are promising targets for remyelinating therapies [15,16,56].

## 5 Conclusions

In summary, this study demonstrates that agathisflavone stimulates oligodendrogenesis and remyelination. A major effect of agathisflavone is the regulation of microglial activation, promoting a microglial phenotype that supports su remyelination and repair. In addition, we show that agathisflavone is neuroprotective and dampens reactive astrogliosis. Finally, we provide evidence that activation of ERα is required for agathisflavone to inhibit microgliosis and promote remyelination. These combinatorial effects of agathisflavone indicate it may be a potential treatment to slow the progression of demyelinating diseases and promote remyelination and repair.

## Declaration of Competing Interest and funding

AMB is a shareholder in the company ‘Glia Genesis Ltd.’. Otherwise, the authors report no conflicts of interest, including personal or financial.

## Author contributions

MMAA performed all experimentation, analyzed, interpreted the data and wrote the manuscript. FP helped to perform immunohistochemistry and confocal microscope images. LMO and MCSJ performed the molecular docking. JMD and JPD performed the chemical analysis and extraction of agathisflavone. VDAS and CSS revised it critically for intellectual content. AMB and SLC supervised the study, edited and reviewed the manuscript. All authors read and approved the final manuscript.
